# Analysis of the psychometric properties of the Sense of Coherence scale (SOC-13) in patients with cardiovascular risk factors: a study of the method effects associated with negatively worded items

**DOI:** 10.1186/s12955-021-01914-6

**Published:** 2022-01-10

**Authors:** Sara Domínguez-Salas, Montserrat Andrés-Villas, Aina Riera-Sampol, Pedro Tauler, Miquel Bennasar-Veny, Antoni Aguilo, Francisco Rivera

**Affiliations:** 1 Department of Psychology, Universidad Loyola Andalucía, 41704 Dos Hermanas, Sevilla Spain; 2 Department of Social, Developmental and Educational Psychology, Faculty of Education Sciences, University of Huelva, 21071 Huelva, Spain; 3 Department of Nursing and Physiotherapy, Research Group On Evidence, Lifestyles & Health (IUNICS), University of the Balearic Islands, 07122 Palma de Mallorca, Spain; 4 Health Research Institute of the Balearic Islands (IdISBa), Palma, Spain; 5 Department of Fundamental Biology and Health Sciences, Research Group On Evidence, Lifestyles & Health (IUNICS), University of the Balearic Islands, 07122 Palma de Mallorca, Spain; 6 Department of Experimental Psychology, Faculty of Psychology, University of Seville, 41018 Seville, Spain

**Keywords:** Sense of coherence, Cardiovascular risk, Method effect, Psychometric analysis, SOC-13

## Abstract

**Purpose:**

The objectives of this study were to analyze the psychometric properties of the Sense of Coherence scale (SOC-13), determine the role of the method effect in the performance of the instrument, and identify the relationship with health perception, quality of life, and sleep quality in patients at cardiovascular risk.

**Methods:**

The final sample consisted of 293 patients at cardiovascular risk, with a mean age of 61.9 years (SD = 8.8), 49.8% of whom were women. The SOC-13, the Patient Health Questionnaire (PHQ-9), and the Medical Outcomes Study-Sleep Scale (MOS-Sleep) were administered. In addition, the participant's self-perceived health and quality of life were also evaluated. All analyses were carried out with SPSS 26.0 and EQS 6.1 statistical software.

**Results:**

The results showed adequate reliability for the SOC-13, with a Cronbach's alpha of .789. The fit of the structures was not adequate in any of the cases (.26 to .62 for one factor, .26 to.73 for three factors, .20 to .54 for one second-order factor, and .25, .42, and .54 for three first-order factors). The three structure models showed an improved fit when adding a latent factor resulting from the method effect (.6 to .85 for one factor, .11 to.90 for three factors, and .11 to .96 for one second-order factor). Moreover, positive correlations were found with health perception, perceived quality of life, and perceived sleep quality.

**Conclusion:**

The SOC-13 is a suitable instrument for patients with cardiovascular risk in Spain, and it is also an indicator of health perception, quality of life, and perceived quality of sleep. Control of the method effect improves the fit of the instrument’s structure. As a future direction, it is recommended to conduct new studies in this and other samples and using different versions of the SOC.

***Trial registration*:**

International Standard Randomized Controlled Trial Number: ISRCTN76069254, 08/04/2015 retrospectively registered.

## Introduction

The Sense of Coherence (SOC) is a central concept of the salutogenic model proposed by Antonovsky in the 1970s [2]. This model shifts the focus from disease to health, well-being, and the resources needed to maintain these. SOC refers to the extent to which a person believes that they can handle the situations they face in different circumstances and moments of their life [56]. It is a construct that includes three dimensions: *comprehensibility*, *manageability*, and *meaningfulness* [1].

For the assessment of SOC, Antonovsky developed a 29-item instrument called the SOC-29 [2]. This instrument evaluates aspects related to the three dimensions that comprise the construct but posits that the instrument has a unidimensional structure rather than a three-dimensional factor structure, which allows for obtaining an overall score instead of one for each dimension [1, 2]. The SOC has been used in at least 49 languages and 48 countries [17]. Furthermore, the instrument has been validated for use in normative and clinical populations with various pathologies [14] and patients with cardiovascular risk or disease [63].

Data from several studies reporting the reliability of the SOC scale scores (in their different versions) have generally found acceptable reliability indicators, which, measured with Cronbach's α, range from 0.70 to 0.95 for the SOC-29 or from 0.70 to 0.93 for the SOC-13 [15, 50, 55].

When developing instruments to assess psychological variables, measures are usually included to control for the acquiescence effect, proposing to include a balanced number of positively and negatively worded items [37]. In this way, the items’ scores are inverted [43], an approach that has been highly recommended [10, 25]. It is assumed that the inversion of the items would not affect the instrument's validity [60]. However, lance and Vandenberg [31] reported that this change in the formulation of the items could affect the instrument's factor structure. In this sense, difficulties in replicating the original structure were found in several instruments when the scale included negative items, which, in exploratory factor analyses, generate a factor that forms part of the variance of those items, a phenomenon known as the method effect [11, 32]. Regarding the SOC, its different versions contain negatively formulated items, which, in many studies, have produced difficulties in replicating its original structure. In this regard, Lin et al. [38] found an improved fit of the SOC-9 when controlling for the method effect.

Regarding the evidence of external validity (referring to the relationship with other variables), the association between SOC and various indicators of health or well-being has been extensively studied. Thus, studies have been conducted to identify the role of SOC in the maintenance or recovery of health. While some reported results show a clear relationship between the SOC and the maintenance of health [33, 47], others consider the instrument as a good indicator of resilience and mental well-being [24, 59], and with a strong influence on the quality of life [16].

Concerning cardiovascular disease (CVD), one of the leading causes of death in Spain [29], studies have identified the role played by habits such as smoking [35], diet [5, 35], alcohol consumption, and physical activity [35] in the prevention or recovery from CVD. Furthermore, although rather less studied, some research suggests the role that a sense of coherence can play in cardiovascular disease. Thus, a relationship has been observed between SOC and quality of life, health perception, or adaptation to the disease, with SOC being understood as a predictor [3, 4, 13, 45, 48, 52, 61]. Thus, a high SOC is related to a better quality of life, better health perception, or better adaptation to the disease. Furthermore, other studies have reported that SOC could be an indicator of psychological well-being, identifying a possible relationship with anxiety and depression [8, 46, 51, 70]. In particular, a high SOC is related to low levels of anxiety and depression. Moreover, regarding the association between SOC and healthy habits relevant to CVD, a positive correlation has been found with diet and physical activity and a negative correlation with alcohol and tobacco consumption [8, 40, 62]. Finally, no validated version is currently available in Spanish for use in patients with CVD.

Considering the above and given the potential the role of SOC in implementing healthy behaviors related to prevention and recovery in noncommunicable diseases, we consider it of interest to determine the suitability of the SOC-13 scale in a Spanish sample of patients at cardiovascular risk. Moreover, we aimed to explore the method effects associated with the instrument's functioning and the relationship between the sense of coherence and other variables such as health perception, perceived quality of life, and sleep quality.

From the review carried out, it is hypothesized that the Sense of Coherence scale will present adequate reliability indicators, and the data will support the classical structures (one global factor or three correlated factors), provided that the method effect is controlled. In addition, it is expected that the data will offer evidence of external validity by showing a high correlation with the other variables studied in the sample of patients with cardiovascular risk.

## Method

### Design

The original study was based on a single-blind, multicenter, randomized clinical trial. However, only the base data were analyzed in this secondary study, so it is considered a multicenter cross-sectional design.

### Participants

The initial sample consisted of 309 patients (49.5% women) with cardiovascular risk factors from primary care centers (urban and rural centers) in Mallorca (Spain). The following cardiovascular risk factors were considered: age (men over 55 years and women over 65 years), presence of hypertension, diabetes, smoking, dyslipidemia and family history, obesity (body mass index > 30 kg/m^2^), and age of onset of cardiovascular diseases.

To participate in this study, participants had to meet the following inclusion criteria: (i) aged between 35 and 75 years, (ii) presence of at least two cardiovascular risk factors, and (iii) presence of cardiovascular risk of up to 15% measured using the Framingham-REGICOR equation. In addition, participants were excluded if: (i) they were institutionalized patients, had a Barthel index below 60, dementia, terminal illness, or cognitive impairment; (ii) showed the presence of myocardial infarction, bypass, or coronary angioplasty in the previous three months, unstable coronary heart disease, or untreated heart failure; (iii) lived outside the healthcare area, and (iv) were participating in another study.

During data processing, it was observed that 16 participants had missing values on the SOC-13 scale and were therefore eliminated, leaving a final sample of 293 participants. Regarding the sociodemographic characteristics of the final sample, 49.8% were men, and the mean age was 61.9 years (SD = 8.8). Regarding marital status, most were in a stable couple relationship or married (75.1%). Concerning educational level, 40.6% of the participants reported having completed primary education, while 22.9% reported having completed lower secondary education and 13.3% upper secondary education. More than half of the sample (53.9%) were retired at the time of participating in the study, while 31.4% were employed.

### Instruments

#### *Sense of Coherence Scale* (SOC-13; [68])

This instrument is a self-administered scale that assesses the sense of coherence as a central concept of the salutogenic model proposed by Antonovsky. This scale is composed of 13 items with seven semantic differential points. The frequency with which participants have certain experiences (e.g., having the impression of being treated unfairly, having confusing feelings or ideas, or being unsure of how to control oneself) is evaluated. The scale's total score ranges from 13 to 91 points and can be used as a single dimension or broken down into three dimensions: meaningfulness, understandability, and manageability. The *meaningfulness* dimension (Items 1, 4, 7, and 12) refers to the value that the person gives to their experiences and their motivation to fight against adversities and challenges in life. The *comprehensibility* dimension (Items 2, 6, 8, 9, and 11) refers to the cognitive capacity to understand and cope with difficult situations. Finally, the *manageability* dimension (Items 3, 5, 10, and 13) represents the person's ability to use the resources available to them effectively.

#### *Patient Health Questionnaire* (PHQ-9; [6])

This self-administered questionnaire constitutes the depression module of the Primary Care Evaluation of Mental Disorders instrument (PRIME-MD [64], that assesses the presence of mental disorders in primary care using the criteria of the Diagnostic and Statistical Manual of Mental Disorders, third edition revised (DSM-III-R) and fourth edition (DSM-IV). The PHQ-9 comprises nine items with a Likert-type response scale ranging from 0 (*Never*) to 3 (*Almost every day*). Participants must indicate how often they have experienced a particular type of distress in the last two weeks through these items. The questionnaire provides a score from 0 to 27 points through the sum of the responses to each item. A higher score is indicative of a greater presence of depressive symptoms. A reliability coefficient of α = 0.792 was obtained for this study.

#### *Medical Outcomes Study—Sleep Scale* (MOS-Sleep; [26])

This is a self-administered questionnaire composed of six items through which six dimensions of sleep are evaluated: (1) initiation, (2) maintenance; (3) quantity; (4) adequacy; (5) somnolence; and (6) respiratory impairments (including shortness of breath or snoring). The participant must respond using a Likert-type response scale ranging from 1 to 5 points for each item. As an outcome measure, and after inverting Items 1 and 6 because they are written in reverse, the sum of the items provides an overall score ranging from 6 to 30 points. A higher score is indicative of greater sleep disturbances. A Cronbach's alpha of 0.743 was obtained for this study.

To assess the participants' self-perceived health and quality of life at the beginning of the study, two ad hoc items were included with a Likert-type response scale with five options (*Very bad, Bad, Fair, Good, and Very good*).

### Procedure

Data were collected as part of a research protocol with International Standard Randomized Controlled Trial Number (ISRCTN): ISRCTN76069254 [54]. The objective of this trial was to analyze the efficacy of a 12-month multifactorial intervention by primary care nurses in increasing adherence to physical activity prescription in patients with two or more cardiovascular risk factors and with cardiovascular risk (determined using the Framingham-REGICOR equation, of up to 15%). Although the patients were evaluated twice—baseline and 12 months after baseline—for the present study, we only considered the data collected at the baseline visit where the SOC evaluation was carried out. Ethical approval has been obtained from the Institutional Review Board of the Balearic Islands Health Service (CEI-IN Ref No.: IB 2341/14).

### Data analysis

First, and considering the distribution of the instruments' scores, univariate normality was assessed using the Kolmogorov–Smirnov test and multivariate normality using the Mardia test. It could not be assumed that this assumption was fulfilled in both cases.

For the item analysis, the item-test correlation was calculated, along with the descriptive statistics and the analysis of each SOC item's floor and ceiling effect. The internal consistency of the total scale and its dimensions were evaluated through the Cronbach's alpha coefficient.

Confirmatory Factor Analysis (CFA) was carried out with the Robust Maximum Likelihood method to study the scale's factorial structure. The following structures were analyzed: (i) one global factor solution, (ii) three-factor correlated solution, (iii) second-order factor with a three first-order factor solution, (iv) one global factor solution with method effect, and (v) three-factor correlated solution with method effect. The indices used to evaluate the model fit were the Satorra-Bentler goodness-of-fit statistic χ^2^ (χ^2^S-B), the Comparative Fit Index (CFI), the Root Mean Square Error of Approximation (RMSEA), the Non-Normalized Fit Index (NNFI), Akaike's Information Criterion (AIC) and Standardized Root Mean Squared Residual (SRMR). CFI and NNFI values above 0.90 are indicative of acceptable fit [44, 58]. However, Hu and Bentler [28] recommend values ≥ 0.95. RMSEA values lower than 0.06 are also indicative of a good fit.

Finally, Spearman correlations were used to study the validity of the SOC-13 scale scores concerning other variables.

All analyses were carried out with SPSS 26.0 and EQS 6.1 statistical software.

## Results

To study the scale's psychometric properties, the results are presented in four sections. First, the scale is analyzed at the item level. Second, the reliability of the scores is analyzed; third, the scale's internal structure is analyzed using CFA as evidence of internal validity, and finally, the relationships with other variables are analyzed as a source of evidence of external validity.

### Item analysis

Table 1 shows the descriptive statistics of the participants’ responses to each of the 13 items that comprise the SOC-13 scale. The item with the highest score was Item 12, "*How often do you have the feeling that there is little meaning in the things you do in your daily life?*" (M = 5.93; SD = 0.09). The lowest score was obtained for Item 11 "*When certain events occurred, have you generally found that: you overestimated or underestimated their importance-you assessed the situation correctly?*" (M = 4.48; SD = 0.13). A ceiling effect was observed in all the scale items, while none presented a floor effect (less than 15% in all cases).

**Table 1:** Descriptive statistics of the SOC-13 scale (N = 293)

Item		Minimum	Maximum	Mean	*SE*	*SD*	Skewness	Kurtosis	% of responses with value 1	% of responses with value 7
1	Do you have the feeling that you really don't care about what is going on around you?	1	7	5.83	0.11	1.83	− 1.401	0.659	4.8	61.8
2	Has it happened in the past that you were surprised by the behavior of people whom you thought you knew well?	1	7	4.65	0.11	1.82	− 0.354	− 0.891	6.8	20.1
3	Has it happened that people whom you counted on disappointed you?	1	7	4.81	0.11	1.87	− 0.465	− 0.945	5.8	24.9
4	Until now, your life has had: no clear goals—very clear goals and purpose	1	7	5.16	0.10	1.76	− 0.764	− 0.339	5.1	30.7
5	Do you have the feeling that you are being treated unfairly?	1	7	5.65	0.11	1.94	− 1.242	0.203	6.8	57.0
6	Do you feel that you are in an unfamiliar situation and don't know what to do?	1	7	5.63	0.11	1.87	− 1.255	0.381	6.5	52.2
7	Doing the things you do every day is: a source of deep pleasure and satisfaction—a source of pain and boredom	1	7	5.46	0.09	1.66	− 0.978	0.182	3.8	37.9
8	Do you have very mixed-up feelings and ideas?	1	7	5.06	0.12	2.10	− 0.665	− 0.979	9.2	41.0
9	Does it happen that you experience feelings that you would rather not have to endure?	1	7	5.33	0.12	1.98	− 0.851	− 0.658	5.8	47.1
10	Many people, even those with a strong character, sometimes feel like losers in certain situations. How often have you felt this way in the past?	1	7	5.23	0.11	1.94	− 0.862	− 0.527	6.8	37.2
11	When certain events occurred, have you generally found that: you overestimated or underestimated their importance-you assessed the situation correctly?	1	7	4.48	0.13	2.27	− 0.262	− 1.493	14	31.7
12	How often do you feel that there is little meaning in the things you do in your daily life?	1	7	5.93	0.09	1.61	− 1.588	1.733	3.8	57.3
13	How often do you have feelings that you are not sure you can control?	1	7	5.81	0.10	1.76	− 1.436	0.927	4.4	56.7

Scale range: 1–7

*SE* standard error, *SD* standard deviation

### Evidence of internal validity: Confirmatory Factor Analysis

**Fig. 1:**
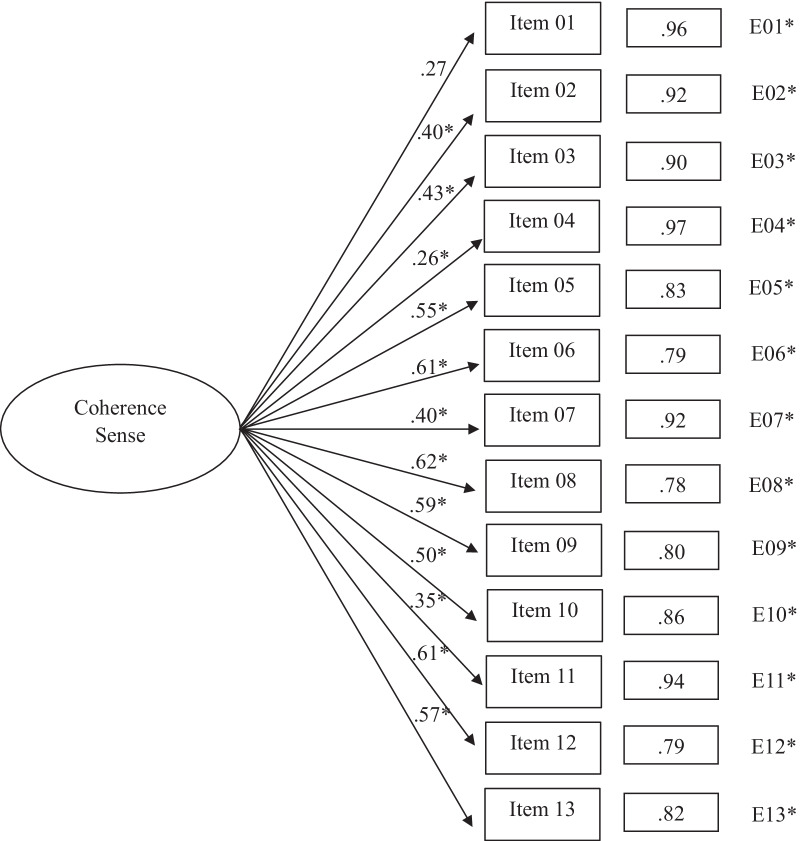
One global factor solution

**Fig. 2:**
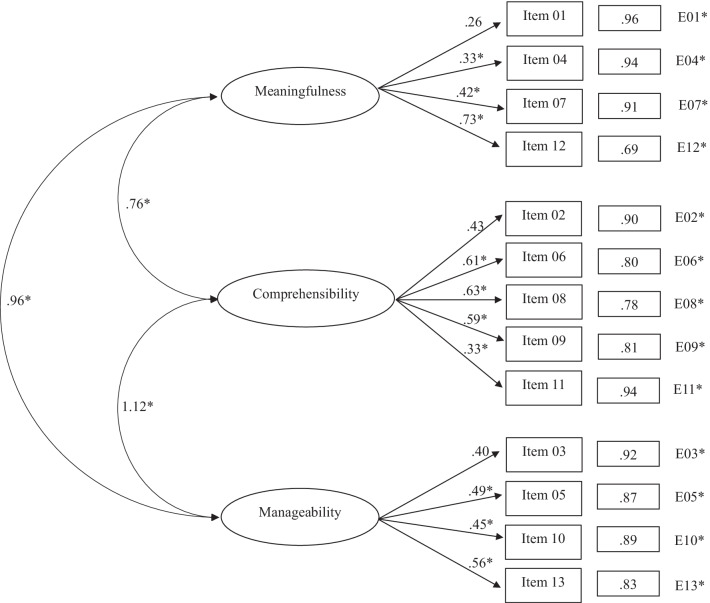
Three factors correlated solution

Table 2 shows the fit indices of the SOC-13 structures subjected to adjustment through the application of CFA. Results of the first model, where a unidimensional structure was proposed, provided inadequate fit indicators. For example, the standardized weights shown in Fig. 1 ranged from 0.26 (Item 4) to 0.62 (Item 8). In a similar vein, the three correlated factors model (*Meaningfulness, Comprehensibility,* and *Manageability*) did not provide a good fit. In this case, values of the standardized loads of the model obtained (Fig. 2) ranged between 0.26 (*Meaningfulness/Item* 1) and 0.73 (*Meaningfulness/Item* 12).

**Table 2:** Fit indices for confirmatory analysis of SOC-13

Model	χ^2^S-B	df	*p*	NNFI	CFI	AIC	RMSEA [95% CI]	SRMR
One global factor solution	339.59	65	< 0.001	0.567	0.639	209.595	0.120 [0.108, 0.133]	0.093
Three factors correlated solution	328.93	62	< 0.001	0.559	0.649	204.935	0.121 [0.108, 0.134]	0.092
Second-order factor with three first-order factors solution	540.41	62	< 0.001	0.209	0.371	416.410	0.163 [0.150, 0.175]	0.206
One global factor solution with method effect	117.13	60	< 0.001	0.902	0.925	− 2.867	0.057 [0.041, 0.072]	0.057
Three factors correlated solution with method effect	99.81	57	< 0.001	0.923	0.944	− 13.775	0.051 [0.034, 0.067]	0.057
Second-order factor with three first-order factors solution with method effect	300.08	57	< 0.001	0.563	0.680	186.085	0.121 [0.107, 0.134]	0.184

χ^2^S–B = Satorra–Bentler scaled Chi-squared test; df = degrees of freedom; p = p value; NNFI = Non-Normalized Fit Index; CFI = Comparative Fit Index; AIC = Akaike's Information Criterion; RMSEA = Root-Mean-Square Error of Approximation; SRMR = Standardized Root Mean Squared Residual

**Fig. 3:**
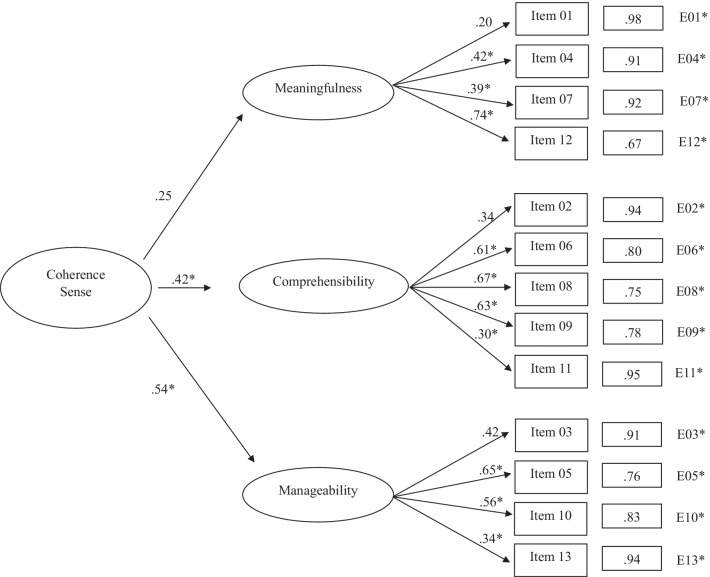
Second order factor with three first order factors solution

In the third model, a structure of SOC-13 composed of a second-order factor with three first-order factors was proposed. The results obtained were χ^2^*S-B* (62) = 540.41; *NNFI* = 0.209; *CFI* = 0.371; *RMSEA* = 0.163 (95% CI 0.150, 0.175). Figure 3 shows that the values of the standardized loads of the model obtained ranged between 0.20 (*Meaningfulness/Item* 1) and 0.74 (*Meaningfulness/Item* 12). The second-order factor showed loads on the factors of *Meaningfulness*, *Comprehensibility,* and *Manageability* of 0.25, 0.42, and 0.54, respectively.

**Fig. 4:**
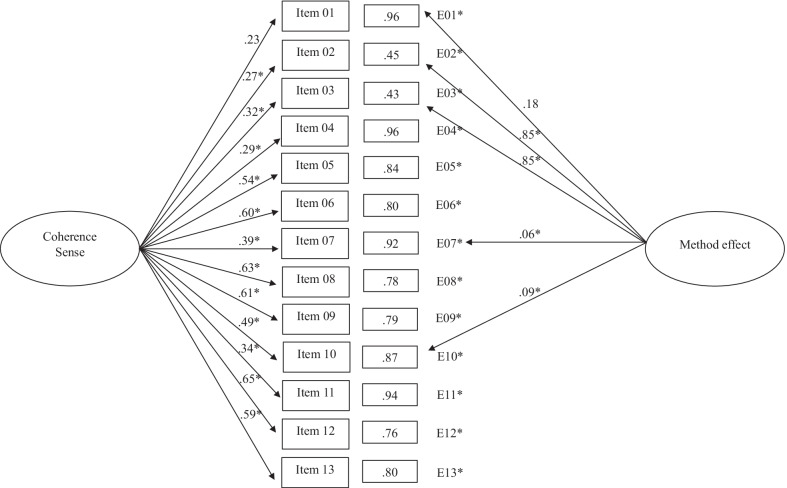
One global factor solution with method effect

After analyzing the classical structures of the SOC-13, we proceeded to study these by adding a latent factor resulting from the method effect, consisting of the negatively worded items (Items 1, 2, 3, 7, and 10). In all cases, the fit indices improved concerning their classical structures (Table 2). Thus, considering the structure of a global factor with the method effect, the results showed adequate fit indices compared to the same structure without the method effect. The standardized method effect weights (Fig. 4) ranged from 0.06 (Item 1) to 0.85 (Items 2 and 3).

**Fig. 5:**
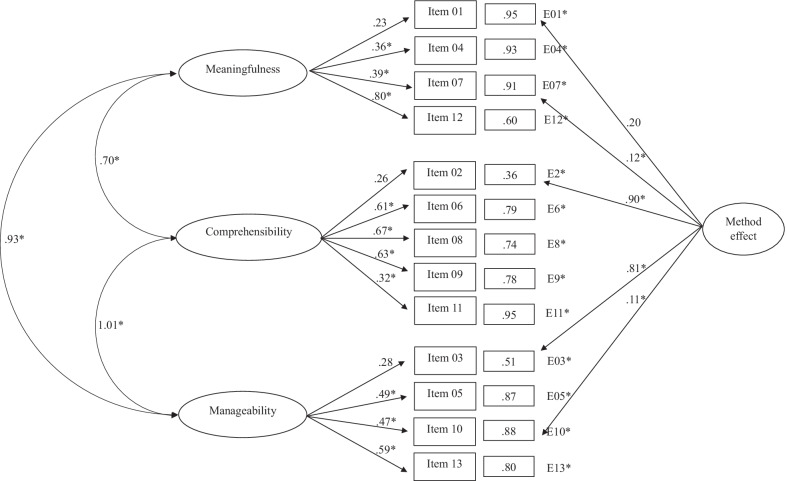
Three factors correlated solution with method effect

The three-factor structure with the method effect provided the best results of all the models analyzed. The results obtained were χ^2^*S-B* (57) = 99.81; *NNFI* = 0.923; *CFI* = 0.944; *AIC* = 186.085; *RMSEA* = 0.051 (95% CI 0.034, 0.067); *SRMR* = 0.057) compared to the same structure without the method effect, the results of which were χ^2^*S-B* (62) = 328.93; *NNFI* = 0.559; *CFI* = 0.649; *AIC* = 204.935; *RMSEA* = 0.121 (95% CI 0.108, 0.134); *SRMR* = 0.092). Figure 5 shows the item loadings of the method effect items, with values ranging from 0.11 (Item 10) to 0.90 (Item 2).

**Fig. 6:**
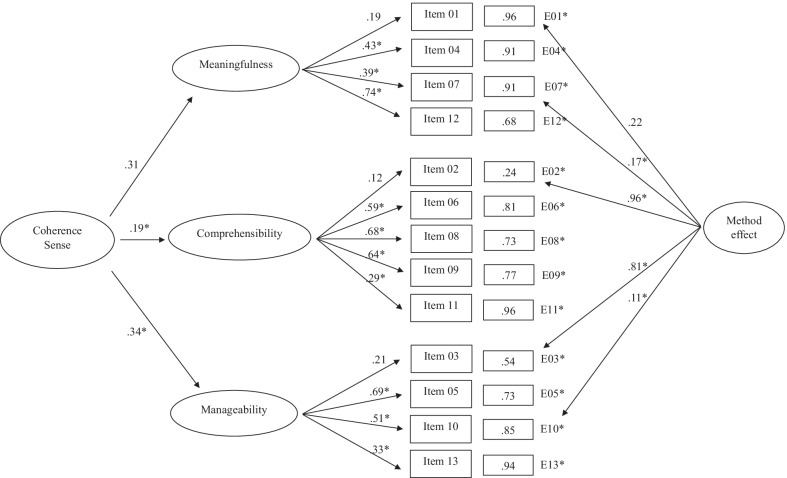
Second order factor with three first order factors solution with method effect

Finally, the structure composed of a second-order factor with three first-order factors with the method effect (Fig. 6) did not provide a good fit.

### Evidence of reliability of scores

Reliability, estimated through Cronbach's alpha internal consistency coefficient, revealed adequate levels with a value of α = 0.789 for the whole scale (Table 3). The reliability coefficients for each dimension were 0.458 for meaningfulness, 0.628 for comprehensibility, and 0.560 for manageability. The item-test correlations ranged from 0.268 to 0.537. Cronbach's alpha values indicated that only the removal of Item 4, *"Until now your life has had: no clear goals—very clear goals and purpose,"* slightly increased the internal consistency of the scale (alpha increment = 0.004).

**Table 3:** Item-test correlations and Cronbach's alpha coefficients (N = 293)

Item	Corrected item-total correlation	Alpha if item deleted
1. Do you have the feeling that you really don't care about what is going on around you?	0.268	0.789
2. Has it happened in the past that you were surprised by the behavior of people whom you thought you knew well?	0.401	0.777
3. Has it happened that people whom you counted on disappointed you?	0.435	0.774
4. Until now, your life has had: no clear goals—very clear goals and purpose	0.207	0.793
5. Do you have the feeling that you are being treated unfairly?	0.477	0.770
6. Do you have the feeling that you are in an unfamiliar situation and don't know what to do?	0.521	0.766
7. Doing the things you do every day is: a source of deep pleasure and satisfaction—a source of pain and boredom	0.359	0.781
8. Do you have very mixed-up feelings and ideas?	0.510	0.766
9. Does it happen that you experience feelings that you would rather not have to endure?	0.504	0.767
10 Many people, even those with a strong character, sometimes feel like losers in certain situations. How often have you felt this way in the past?	0.437	0.774
11. When certain events occurred, have you generally found that: you overestimated or underestimated their importance-you assessed the situation correctly?	0.323	0.787
12. How often do you have the feeling that there is little meaning in the things you do in your daily life?	0.537	0.767
13. How often do you have feelings that you are not sure you can control?	0.479	0.770

### Evidence of external validity based on the relationship with other variables.

Regarding validity in relation to other variables, the SOC-13 scores correlated significantly and negatively (and with a large effect size) with the PHQ-9 (rho = -0.543) and with a medium effect size with the MOS-Sleep scale (rho = -0.388). Thus, higher levels of SOC were associated with a lower presence of depressive symptoms and sleep disturbances. Similar results were obtained with the dimensions of the SOC-13 (Table 4).

**Table 4:** Correlation between SOC-13 and related constructs

	MOS-Sleep	PHQ-9	Self-perceived health	Self-perceived quality of life
SOC-13 Total	− 0.388**	− 0.543**	0.146*	0.236**
Meaningfulness	− 0.298**	− 0.387**	0.043	0.177**
Comprehensibility	− 0.330**	− 0.471**	0.172**	0.222**
Manageability	− 0.347**	− 0.473**	0.144*	0.203**

MOS-Sleep: Medical Outcomes Study-Sleep Scale; PHQ-9: Patient Health Questionnaire

*p < 0.005; **p < 0.001

Concerning participants' self-perceived health, significant and positive correlations were obtained with the SOC-13 total score (rho = 0.146) and with the *comprehensibility* (rho = 0.172) and *Manageability* (rho = 0.144) dimensions, all with a small effect size. Finally, self-perceived quality of life was also significantly and positively related to SOC-3 (rho = 0.236) and its dimensions (Table 4), with a small effect size in all cases.

## Discussion

The results of this study show adequate reliability of the SOC-13 in patients with CVD, with a Cronbach's alpha of 0.789, which is within the range of values (0.70–0.93) obtained in previous studies [2, 15, 50, 53]. The reliability indicators of the three dimensions were also similar to those reported by other authors [12, 39, 66, 68]. Despite being adequate, the low reliability of the scale or the dimensions prompted a detailed analysis of the weight of the items and the effect of their elimination, observing that the elimination of some of these improved the fit of the instrument. The items most frequently identified in this regard were Items 5 and 6 [9, 36]. However, in agreement with results from the present study, it has previously been suggested that the elimination of Item 4 also results in an improved fit of the instrument [49]. Our results indicate that the elimination of Item 4 produced a slight increase in internal consistency, from 0.789 to 0.793.

Regarding the instrument’s structure, previous studies have yielded mixed results. Thus, data point to a one-factor structure [1, 2, 9, 21, 22, 27, 30] or a three-factor structure [23, 34, 67, 71], while other studies report evidence of two factors [57], or a structure of three secondary factors and a primary global factor [18–20]. Similarly, some studies have found different factorial structures, such as one with seven first-order factors and two second-order factors or a four-factor structure [7, 41, 68, 69, 71]. In our case, the scale structure did not show a good fit to any of the assumptions made. The considerable diversity in the factors across different versions, particularly the SOC-13, has prompted us to think about the elimination of items [9, 36] or deficiencies related to the structure of the instrument [55]. In an attempt to address and find a solution to this deficiency, several authors have proposed conducting factor analyses in search of other structures [7, 41, 68, 69, 71]. Although more adequate structures have been reported, they have not been replicated in other studies. On the other hand, and taking into account the presence of items with a negative formulation, following the suggestion of Lin et al. [38], we propose to analyze the influence of this formulation on the internal structure of the instrument by controlling for the method effect in the CFA. Results obtained by Lin et al. [38] regarding the SOC-9 showed an improved fit of the one-factor structure, but this was not the case with the three-factor model. The present study shows how the instrument fit improves in the three models studied, finding that the greatest changes are found for the correlated three-factor structure. These findings lead us to suggest that the inversion of items, or the use of negatively worded items, may have been one of the factors that contributed to the discrepant results found when the structure of the instrument (method effect) was analyzed. Therefore, the method effect should be considered in future studies, adjusting the scale to minimize its impact.

The external validity analyses show that the SOC-13 score in patients with CVD is positively related to health perception, perceived quality of life, and sleep quality, as predicted by the literature. These data are in agreement with results of previous studies conducted in different populations, in which similar relationships have been reported between SOC-13 and the variables indicated above [7, 16, 33, 42, 47, 65, 68]. In addition, and specifically in populations from other countries with cardiovascular risk factors, a relationship has already been identified between the SOC-13 [13, 52] and quality of life, as well as the SOC-13 and health perception [4].

Therefore, the data suggest that in patients at cardiovascular risk, the SOC can be understood as a helpful indicator for predicting other variables such as health perception, perceived quality of life, and sleep quality. Nonetheless, to confirm that SOC can serve as an adequate predictor of these variables, it will be necessary to carry out studies that determine the variation shown by all these variables over time.

## Strengths and limitations

The main strength of the study is that it is the first version of the SOC validated in Spanish for use in patients with cardiovascular disease. In addition, only one previous study has analyzed the method effect in the SOC-9, and our study goes a step further to show how controlling for the method effect improves the fit of the SOC-13. Further strengths are the clinical sample size and the validity of the instruments used to measure the variables of interest.

One of the limitations of this study is the lack of longitudinal data that would allow us to analyze the evolution of the relationship between SOC-13 and perceived quality of life, health perception, and sleep quality. Thus, we cannot draw any firm conclusions regarding the extent to which SOC can serve as a predictor variable.

## Conclusions

In conclusion, the SOC-13 scale is suitable for use in Spanish patients at cardiovascular risk, with adequate reliability indicators. In terms of internal structure, we conclude that the formulation of negative items may be responsible for the lack of fit to the classic structures. Furthermore, the results show that controlling for the method effect improves the fit in all cases, with the three-factor structure showing the greatest increase in fit. Finally, we can conclude that the SOC-13 could be an adequate indicator of health perception, perceived quality of life, and sleep quality in Spanish patients at cardiovascular risk.

As a future direction, we recommend that further studies are conducted with this and other versions of the SOC and other clinical and normative samples.

In short, the SOC, as proposed by Antonovsky [2] in his salutogenic model, appears to be a useful instrument for identifying how people adapt to various life situations.

## Data Availability

The data that support the findings of this study are available from the corresponding author, [MAV], upon reasonable request.

## Abbreviations

AIC: Akaike's Information Criterion
CFA: Confirmatory Factor Analysis
CFI: Comparative Fit Index
CVD: Cardiovascular disease
DSM-III-R: Diagnostic and Statistical Manual of Mental Disorders, third edition, revised
DSM-IV: Diagnostic and Statistical Manual of Mental Disorders, fourth edition
ISRCTN: International Standard Randomized Controlled Trial Number
MOS-Sleep: Medical Outcomes Study-Sleep Scale
NNFI: Non-Normalized Fit Index
PHQ-9: Patient Health Questionnaire
PRIME-MD: Primary Care Evaluation of Mental Disorders
RMSEA: Root Mean Square Error of Approximation
SE: Standard error
SD: Standard deviation
SOC: Sense of Coherence
SOC-13: Sense of Coherence Scale
SRMR: Standardized Root Mean Squared Residual
χ^2^S-B: Satorra Bentler scaled Chi-squared test
